# Dr. Barrett’s Statement

**Published:** 1892-09

**Authors:** C. W. Stainton

**Affiliations:** 37 Court Street, Buffalo, N. Y.


### Domestic Correspondence.

### DR. BARRETT'S STATEMENT.

[We regarded the controversy in connection with the new department of dentistry of the University of Buffalo as having been closed in our last number, but those in opposition to the methods employed claim to be heard. In view of this not unreasonable request, we admit the following communication.—Ed.]

The "statement" of Dr. Barrett in the International Dental Journal for August properly demands a reply. The question involved is a new one; at least it has not been presented in its present aspect before. There can be no difference of opinion on this point.

Another equally clear and undisputed statement would be, that if this action of the University of Buffalo, in starting its dental department, is proper and free from objections, then any university in the United States can originate a dental department any time it considers it for its interest so to do, and will have no difficulty in finding men who will accept at once honorary degrees and positions in the new school. In that case the dental profession would really have no control over educational institutions. We are suffering now from a "craze" to start dental schools.

Dr. Ottofy's report, as chairman of the Section on Dental Education, etc., at the recent meeting of the American Dental Association, shows that the total number of graduates of some twelve of our schools was less than one hundred. Surely the need of more schools is not apparent.

We have arrived at a time in our history when we can scrutinize not only the character and standing of our schools, but also their origin and antecedents. The University of Buffalo is undoubtedly an institution of character and standing. There is no disposition to belittle it in any quarter. Surely, however, it has no "divine rights." Any action on its part is of human origin, and subject to fair discussion and frank criticism.

The action had by the University is contrary to the present trend of thought in the dental world. This ought to have been known to some of those interested in the new school. How such action could have been had in ignorance, in view of what has been said and done, is very difficult to comprehend. That the first impression every intelligent dentist had of it was unfavorable cannot be denied.

So far as I am aware, the by no means easy task of defending and explaining this action has fallen upon Dr. Barrett; at least he is the only champion of the University that has prominently presented.

Dr. Barrett is a fluent speaker and writer; in well-rounded periods he excels. But, like many free writers, he oftentimes makes sad havoc with his logic.

The force and weight of what any man says or writes depends not alone on the language used, but upon general accessory facts and considerations. For instance, if a statement made by an individual is in direct contradiction to previous views promulgated by the same writer on the same subject, the strength and force of the statement is very materially diminished; and this is the difficulty with the communication of Dr. Barrett in the August issue. He here earnestly endeavors to justify the conferring of honorary degrees.

This is contradictory to his views on the same subject as expressed in the last January number of the *Dental Practitioner and Advertiser*, in proof of which the following extract is given:

"*Professor Taft speaks approvingly of the recognition of eminent attainments by the conferring of honorary degrees. Therein is found the essence of the whole matter. What authority have schools to recognize merit any more than any other body? A diploma should be a certificate of study, and it is a fraud when it represents anything else. The Delavan diplomas were what Professor Taft approves,—'marks of recognition of eminent services.' The whole system of conferring diplomas as marks of honor is wrong. True, it exists, and has long existed; but so have measles and bum-bailiffs. This pronouncing upon who is and who is not entitled to honor is as foreign to the province of the schools as was the Pope's bull against the comet foreign to religion. A college diploma is a certificate of study,—or it ought to be,—and the man who has not pursued the curriculum of study of the school is no more entitled to it than he is to the tail of the monkey.*"

The statement made that these degrees were conferred for the purpose of expressing the good-will of the University towards dentistry, and to signalize the formation of its new department, is not sustained by the facts.

That the degrees were conferred as a *qualification* was admitted to me by one of the recipients before the event, and by the others subsequently.

That the "University conferred these honorary degrees in perfect good faith and without the slightest thought of doing anything that would subject itself to criticism," can hardly be true, for both recipients of this degree and several of the main movers in the matter in the University were notified in writing, some weeks before the action was had, that if such were adopted the attention of the National Association of Dental Faculties would be called to it, and that the recognition of the school would thusbe prevented.

Dr. Barrett speaks of the New York State degree as being the *highest* qualification that any man can hold in that State. In January he speaks of it as "our bastard State degree,—one that has neither legitimate educational father or mother, and which does not pretend to be an evidence of having pursued any course of study whatever."

C. W. Stainton.

37 Court Street, Buffalo, N. Y.

